# *Streptomyces venezuelae* ISP5230 Maintains Excretion of Jadomycin upon Disruption of the MFS Transporter JadL Located within the Natural Product Biosynthetic Gene Cluster

**DOI:** 10.3389/fmicb.2017.00432

**Published:** 2017-03-21

**Authors:** Stephanie M. Forget, Jennifer McVey, Leo C. Vining, David L. Jakeman

**Affiliations:** ^1^Department of Chemistry, Dalhousie UniversityHalifax, NS, Canada; ^2^Department of Biology, Dalhousie UniversityHalifax, NS, Canada; ^3^College of Pharmacy, Dalhousie UniversityHalifax, NS, Canada

**Keywords:** natural products, MFS transporters, streptomyces, drug efflux, jadomycins, major facilitator superfamily

## Abstract

JadL was identified as a Major Facilitator Superfamily (MFS) transporter (T.C. 2.A.1) through sequence homology. The protein is encoded by *jadL*, situated within the jadomycin biosynthetic gene cluster. JadL has, therefore, been assigned a putative role in host defense by exporting its probable substrates, the jadomycins, a family of secondary metabolites produced by *Streptomyces venezuelae* ISP5230. Herein, we evaluate this assumption through the construction and analysis of a *jadL* disrupted mutant, *S. venezuelae* VS678 *(*Δ*jadL::aac(3)IV)*. Quantitative determination of jadomycin production with the *jadL* disrupted mutant did not show a significant decrease in production in comparison to the wildtype strain, as determined by HPLC and by tandem mass spectrometry. These results suggest that efflux of jadomycin occurs upon disruption of *jadL*, or that JadL is not involved in jadomycin efflux. Potentially, other transporters within *S. venezuelae* ISP5230 may adopt this role upon inactivation of JadL to export jadomycins.

## Introduction

**Figure 1:**
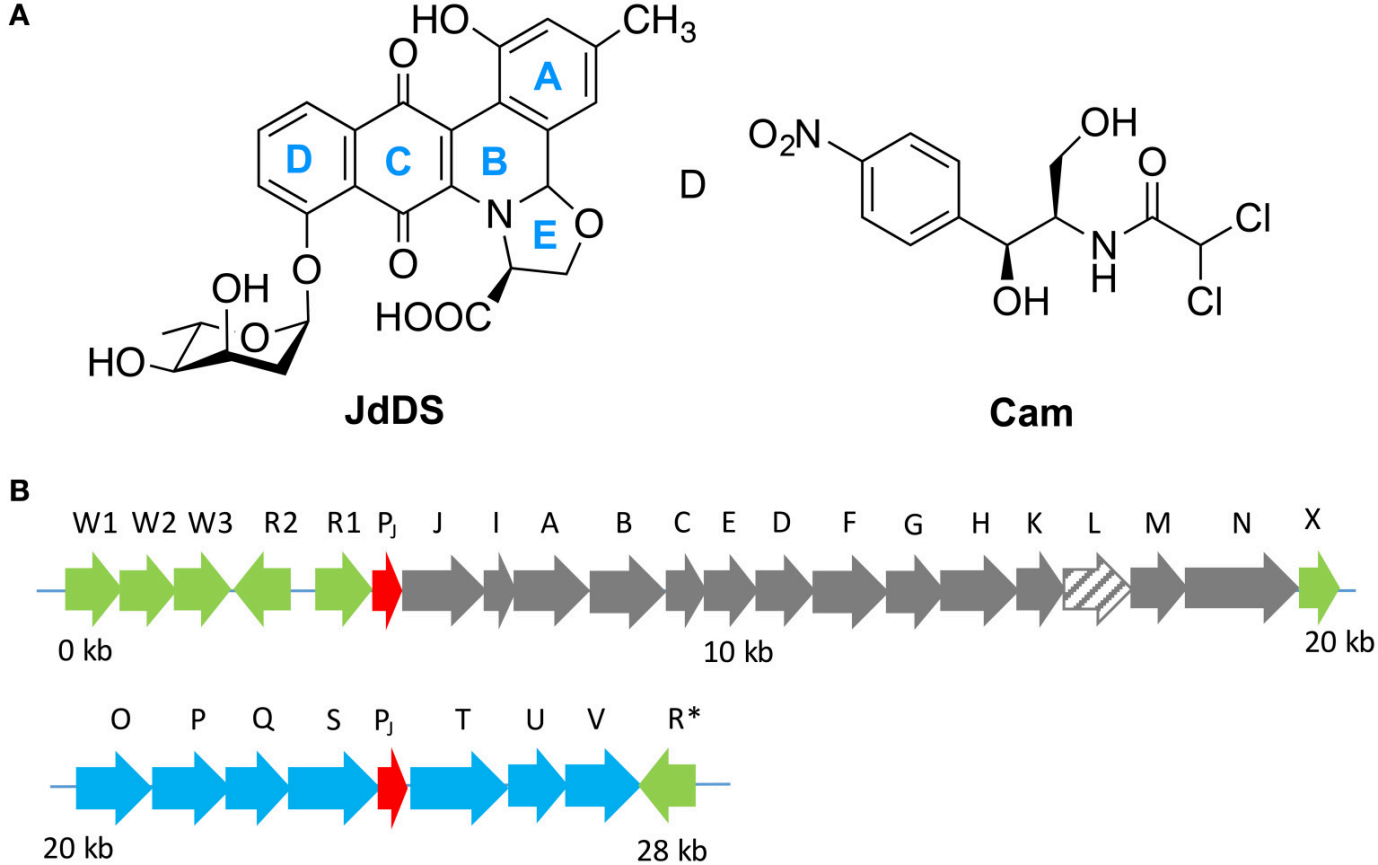
**(A)** Extensive cross regulation has been reported between the biosynthetic pathways of jadomycins (i.e., **JdDS**) and chloramphenicol (**Cam**) within *S. venezuelae* ISP5230. **(B)** The jadomycin biosynthetic gene cluster. Regulatory genes are colored in green, predicted promoter regions in red, angucycline biosynthetic structural genes in gray, sugar biosynthetic structural genes in blue, and jadL, which codes a predicted transport protein, is hatched.

Secondary metabolite production is controlled by complex regulatory networks which are affected by environmental factors such as temperature, nutrient availability, and signaling. Given the complex nature of such regulatory systems, the expression of secondary metabolites is often difficult to replicate within a laboratory setting, and many clusters remain silent (Rutledge and Challis, 2015). The jadomycins (Figure 1A) are angucycline antibiotics (Ayer et al., 1991; Doull et al., 1993) with cytotoxic activities (Jakeman et al., 2009a; Dupuis et al., 2012) and unique drug efflux properties in drug resistant breast cancer cell lines (Issa et al., 2014; Hall et al., 2015) produced by *Streptomyces venezuelae* ISP5230 (ATCC10712) that are regulated by a “cryptic” pathway; expression is induced with the use of minimal media under stress conditions including ethanol shock, phage induction or co-culture with yeast (Doull et al., 1994; Jakeman et al., 2006). In the absence of these additional stress factors, chloramphenicol (**Cam**) is the major natural product produced by *S. venezuelae* ISP5230. The E-ring present in the jadomycin angucyclic framework arises from a rare spontaneous biosynthetic step involving the incorporation of an amino acid. This chemistry enables a strategy for facile derivatization of the jadomycins using culture media containing a single amino (Jakeman et al., 2005, 2009b; Robertson et al., 2015). Recently, as a result of genome analysis a number of natural products have been discovered from *S. venezuelae* ISP5230, including gaburedin (Sidda et al., 2014), venezuelin (Goto et al., 2010), forxymithine (Kodani et al., 2015), (+)-isodauc-8-en-11-ol (Rabe et al., 2015), and venemycin (Thanapipatsiri et al., 2016).

**Figure 2:**
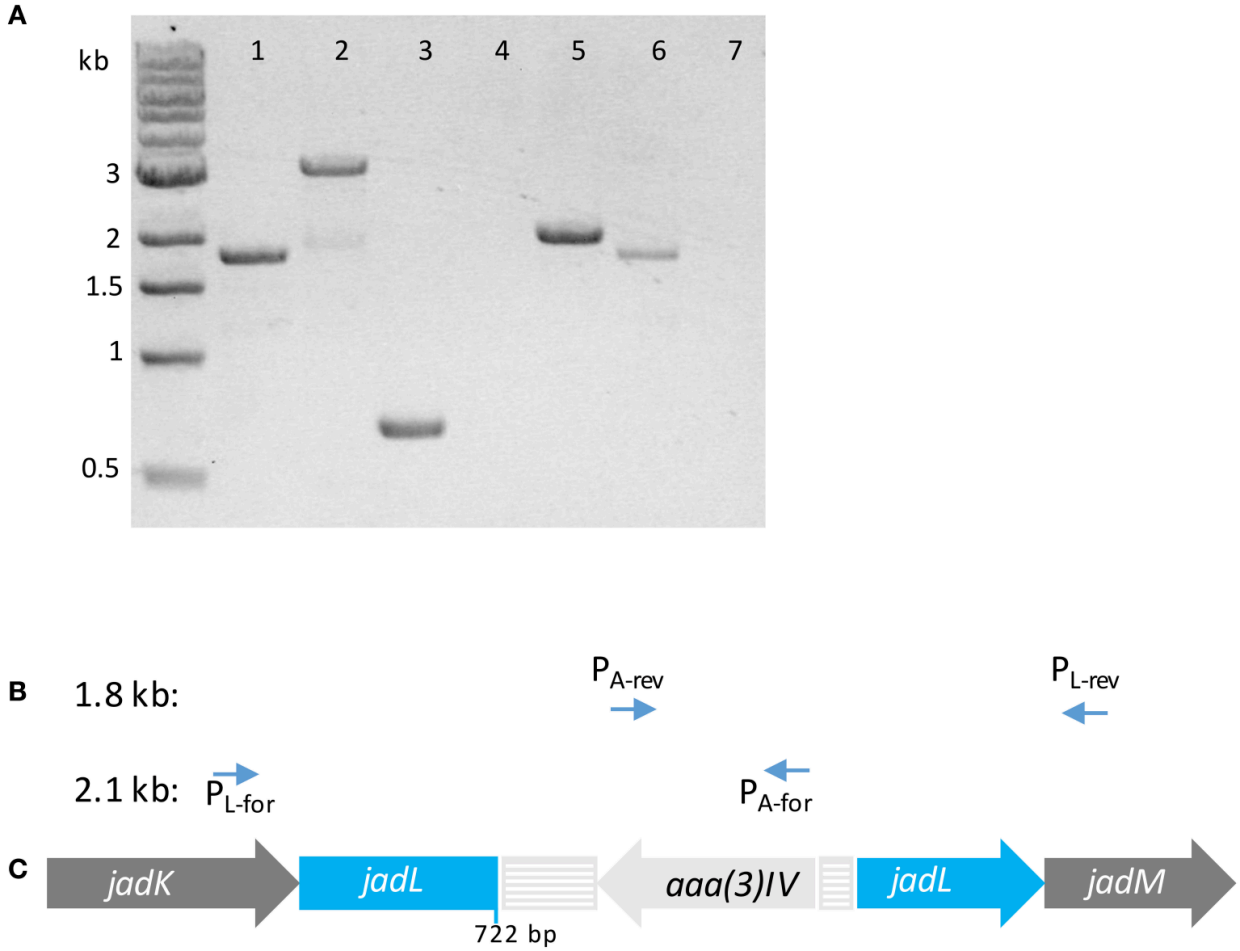
**(A)** Tris-acetate-EDTA (TAE) agarose gel (1% *w/v*) of PCR products using template *S. venezuelae* ISP5230 gDNA (lane 1) and *S. venezuelae* VS678 gDNA (lanes 2–7) with indicated primers (1) P_L-for_/P_L-rev_; (2) P_L-for_/P_L-rev_; (3) P_Apr-for_/P_Apr-rev_; (4) P_L-for_/P_Apr-rev_; (5) P_L-for_/P_Apr-for_; (6) P_L-rev_/P_Apr-rev_; (7) P_L-rev_/P_Apr-for_. Successful amplification of fragments in lanes (5) and (6) indicate that the apramycin resistance gene (*aaa(3)IV*) within the resistance cassette is coded on the 3′–5′ strand. **(B)** Primers pairs that amplified products of the indicated size. **(C)** The orientation of the resistance cassette containing *aaa(3)IV* in *S. venezuelae VS678* as deduced from the PCR products. The disruption casette is colored in pale gray and the disrupted gene (*jadL*) is colored in blue.

JadL is a putative efflux protein coded within the jadomycin biosynthetic gene cluster (Figure 2). JadL is a member of the Major Facilitator Superfamily MFS, Transporter Classification (T.C. 2.A.1), a large and functionally diverse, although structurally and mechanistically conserved, family of transporters with substrates including sugars, amino acids, peptides, drugs, and small anions or cations (Quistgaard et al., 2016). Given the location of *jadL* within the context of the jadomycin gene cluster, we predicted that its primary role would be extracellular export of jadomycins. Herein, we have studied the effect of *jadL* disruption on jadomycin production, that we hypothesized would result in a reduction in the amount of excreted jadomycin.

## Materials and methods

### Bioinformatics tools

BLAST searches were performed using the standard BlastP (**RRID:SCR_001010**) program with the JadL amino acid sequence as input (GenBank: CCA59275.1). Searches excluded environmental and non-cultured strains. BlastP searches were also conducted against the data set from the Transporter Classification Database (TCDB, **RRID:SCR_004490**). Predictions for transmembrane domains were performed using the constrained consensus TOPology prediction server (CCTOP, http://cctop.enzim.ttk.mta.hu).

### Strain maintenance and growth

*Streptomyces venezuelae* ISP5230 (ATCC10712) spores were maintained as solutions in 25% glycerol at −70°C (Kieser et al., 2000). For natural product productions, *S. venezuelae* strains were maintained at 30°C on maltose yeast malt extract (MYM, maltose 0.4% w/v, yeast extract, 0.4% w/v, malt extract 1% w/v, pH 7.0) agar for 1–2 weeks before inoculation in MYM broth. Media was supplemented with 50 μgmL^−1^ apramycin for disruption strains.

### Construction of the *jadL* disruption mutant: *S. venezuelae* VS678 *(ΔjadL::aac(3)IV)*

A 4 kb DNA fragment containing *jadL*, a SacI digest product from lambda clone LH7 (Han et al., 1994), was ligated into SacI linearized pHJL400 (Larson and Hershberger, 1986) to give pJV105A and pJV105B, in which inserts are oppositely oriented. pJV105A was digested with NcoI, situated 722 bp after the *jadL* start codon, and ligated with an apramycin resistance cassette bearing flanking NcoI sites to produce the disruption (*jadL*::*aaa(3)IV*) vector pJV106. *S. venezuelae* ISP5230 protoplasts, prepared as described elsewhere (Yang et al., 1995; Kieser et al., 2000), were transformed with pJV106, regenerated on R5N agar (Aidoo et al., 1990) and overlaid with soft nutrient agar containing thiostrepton (25 μgmL^−1^) after 10 h. Transformants were patched to MYM agar containing thiostrepton (25 μgmL^−1^) then replica plated to MYM containing apramycin (50 μgmL^−1^). Single crossover mutants, resistant to both thiostrepton and apramycin, were patched to MYM containing apramycin and carried through three rounds of sporulation. After this time, colonies with the appropriate phenotype for double crossover mutants, i.e., resistance to apramycin and sensitivity to thiostrepton, were screened by PCR. PCR screening to confirm insertion of the apramycin disruption cassette used a primer pair flanking *jadL*: P_L-for_ 5′-ACCTTCGCCGAGTACGAGTC-3′ and P_L-rev_ 5′-TGTGCGACAGCGAGAAG-3′. Primers used to determine the orientation of the apramycin resistance gene were P_Apr-for_ 5′-TGCTGGTCCACAGCTCCTTC -3′ and P_Apr-rev_5′-GAGCGGCATCGCATTCTTC-3′. All PCR reactions were performed using Phusion DNA polymerase (New England Biosciences) and were supplemented with 10% dimethyl sulfoxide with reaction conditions following manufacturer protocols. Template genomic DNA was isolated from overnight *S. venezuelae* cultures (MYM, 30°C, 250 rpm) using standard commercial kits.

### Culture conditions for jadomycin DS (JdDS) production

Standard jadomycin production protocols were followed (Jakeman et al., 2006) with d-serine as sole amino acid in the production medium ensuring biosynthesis of JdDS. A 1 cm^2^ patch of cells was harvested from 1 to 2 week old MYM-agar plates, with 50 μgmL^−1^ apramycin for disruption strains, and used to inoculate MYM without antibiotics. The inoculum was shaken (250 rpm) at 30°C for 18–20 h. The cells were then harvested (5,000 rpm), the supernatant decanted, and the cells washed with minimal salt media [MSM, MgSO_4_ 0.4 g/L, MOPS 1.9 g/L, salt solution (1% w/v NaCl, 1% w/v CaCl_2_) 9 mL/L, FeSO_4_-7H_2_O (0.2% w/v stock solution) 4.5 ml/L, trace mineral solution (ZnSO_4_·7H_2_O 880 mg/L, CuSO_4_·5H_2_O 39 mg/L, MnSO_4_·4H_2_O 6.1 mg/L, H_3_BO_3_ 5.7 mg/L, and (NH_4_)_6_Mo_7_O_24_·4H_2_O 3.7 mg/L) 4.5 ml/L, pH 7.5] twice. Cells suspended in MSM were used to inoculate fresh MSM supplemented with d-serine (60 mM), dextrose (33 mM), and phosphate (50 μM) to an initial OD_600_ ~0.6. Ethanol was added to the culture media to a final concentration of 30 μL per 1 mL media. Ethanol shocked cultures were shaken (250 rpm) at 30°C for 52 h. Cell growth was monitored by measuring the OD_600_ at the indicated time intervals by withdrawing 600 μL aliquots from each of triplicate cultures; A_526_ measurements were recorded using the corresponding clarified aliquots. Absorbance data was plotted with GraphPad Prism 6.02 software.

### Isolation of JdDS and analysis of natural product profile

All solvents used for natural product purification were high performance liquid chromatography (HPLC)-grade. After incubation for 52 h, jadomycin production cultures (50 mL) were pelleted by centrifugation (8,000 rpm). The supernatant was passed through 0.22 μM Millipore filters. Clarified culture media was passed through a 2 g Silica-phenyl column (Si-Ph, Silicycle) to which the jadomycins bind tightly. The column was washed with water, until the flow through was colorless, and HPLC-grade methanol was used to elute compounds bound to the column. After concentration by evaporation (Genevac EZ-Bio personal evaporator), the mass of the crude extract was determined. Analysis of the natural products produced by each strain was performed by analytical HPLC, using a previously described method (Robertson et al., 2016), and by thin layer chromatography (TLC), using glass-backed silica plates plates (SiliCycle, 250 μM, F_254_ silica) and 5:5:1 ethyl acetate: acetonitrile:water as the eluent. Crude material was solubilized in methanol for TLC analysis. Visualization reagents were not required as jadomycins are deeply colored.

### Quantification of JdDS by LCMS^2^

The concentration of **JdDS** produced by each of the wildtype and *S. venezuelae* VS678 strains was quantified by liquid chromatography coupled to mass spectroscopy (LCMS). A purified sample of **JdDS** was used to construct a calibration curve. Electrospray ionization in positive mode (ESI^+^) LCMS experiments were run using an HPLC (Agilent 1100) equipped with a reversed phase column (Phenomenex Kinetic 2.6 μM Hilic, 150 × 2.1 mm) coupled to a hybrid triple quadrupole mass spectrometer (Applied Biosystems, 2,000 Qtrap). LCMS instrumentation and running conditions have been described elsewhere (Robertson et al., 2016). The following settings were applied for the acquisition of enhanced product ion scan (EPI) experiments: capillary voltage +4,500 kV, declustering potential +80 V, and curtain gas 10 (arbitrary units). To construct the standard curve, EPI experiments with 5 μL injections of purified **JdDS** at 2, 5, 10, and 15 μM in methanol were collected with m/z [M+H]^+^ m/z 524 as the parent ion. In the resulting spectra, the area beneath the peak for the parent ion at m/z 524 and for the fragment ion at m/z 394 were determined by integration using the LCMS software (Applied Biosystems, Analyst version 1.4.1). The relationship between **JdDS** concentration (μM) and area (unitless) was solved using linear regression. Crude samples (from the Si-Ph column methanol extract) were taken up in 1 mL methanol, then diluted 200-fold by serial dilution. After LCMS^2^ analysis, the intensities under the m/z 524 and 394 peaks were used to determine the concentration of **JdDS** using the linear relationship described above. An average of the values obtained from each curve was used to calculate the total amount of **JdDS** per 50 mL bacterial culture.

## Results and discussion

### Predicted properties of Jadl based on sequence homology

The gene, *jadL*, is found amongst the structural genes within the jadomycin biosynthetic gene cluster (Han et al., 1994; Wang and Vining, 2003; Pullan et al., 2011). The gene encodes a protein with 459 amino acids that contains an MFS_1 and an H^+^ antiporter domains. The domains identified suggest an antiporter mode of substrate transport reliant on a proton gradient. MFS family transporters are ubiquitous; within the genome of *S. venezuelae*, a Pfam search for the MFS_1 domain identified 106 sequences. As is the case for most members of the MFS superfamily, JadL is predicted to have 12 transmembrane (TM) domains (Table 1), with the *N*- and *C*-terminal domains each comprising of 6 TM domains. The position of the disruption cassette begins at amino acid position 241 (from the N-terminus) and is situated at the beginning of predicted TM helix 7, which positions the disruption cassette between the *C*- and *N*-terminal domains. It is widely accepted that MFS family transporters have highly conserved structures and operate by the same “clamp and switch” mechanism (Quistgaard et al., 2016). MFS family transporters are active in a monomeric form, where the substrate binding occurs at the cleft between the *N*- and *C*-terminal domains; substrate and proton binding is mediated by a number of amino acids contacts located on the TM regions scattered over both the *N*- and *C*-terminal domains (Quistgaard et al., 2016). Such interactions have been demonstrated in several crystal structures, a few of the many examples include the well-studied *Escherichia coli* lactose permease (LacY) (Abramson et al., 2003), and multidrug transporters such as *E. coli* ErmD (Yin et al., 2006) and MdfA (Heng et al., 2015). Thus, we predict that the *jadL* disruption mutant will be unable to bind or transport its substrate(s).

**Table 1:** **JadL protein sequence with TM regions (CCTOP) shown in bold**.

MVKARSNTFRSLSVRNFR**LFAAGQVVSVAGTWTMVVA**QDWLVLGMTGDSGT**AL** **GAVTALQFAPMLLLTLYGGR**LADRYDKRMLLTAAN**LTAGALAAVLAVLVLT**GG**VR** **LWHIWLLALGIGVVNAV**EVPTRMSFVGELVGNELLPNASAL**SAAYFSVARVAGPA** **LAGLLITG**FG**TGWAIALNAVSYLATVAGLRMMRPEENPGGARGGRPEAGQGAR** **KEERKDARVVDGLRYTASRADLTLPMALVAVIGLCGMNFQLTLPLLAKTVFHADA** **TSFGLLTTAFAAGSLLGAIAG**TRRSGRPAA**RTVIGSALAFGALEAAAGW**AP**GFL** **FAVVLLTLTGFASIYFAQA**ANHRIQLGSDPAYRGR**ILALYTLILQGSTPLGALLVGL** LTERLGAR**AGLWLGGLVSLAAALVALGL**EYRGTRPARTAAAPDPSRGPDSDSPD PDSDPDSRERLVRDAAPEGRGR

In a query for genes homologous to *jadL* a BlastP search identified *kinJ* (69% identity), a gene found within the kinamycin, a diazo-containing glycosylated angucycline, biosynthetic pathway of *Streptomyces murayamaensis* (Gould et al., 1998). The top protein blast hits, all uncharacterized MFS family transporters from actinobacteria with high identity similarities (67–71%), are listed in Table 2. A BlastP search against the TCDB is summarized in Table 3. The majority of the homolog identified (20–30% identity) were from family 2.A.1.21, the drug: H+ antiporter-3 (12 spanner) (DHA3) family, which is consistent with the hypothesized role of JadL. Members of the 2.A.1.38, the enterobactin (siderophore) exporter (EntS) family, and 2.A.1.30, the putative abietane diterpenoid transporter (ADT) family, were also amongst the top hits from the TCDB database.

**Table 2:** **Top 10 BlastP results for JadL**.

**Hit**	**Protein accession**	**Organism**	**Amino acid length**	**Identity**
1	OAR23019	*Streptomyces* sp. *ERV7*	429	67
2	AAO65354	*Streptomyczs murayamaensis*	426	69
3	KPH97664	*Actinobacteria bacterium OK006*	454	68
4	WP_060900228	*Streptomyces diastatochromogenes*	418	68
5	WP_053676855	*Streptomyces* sp. *WM4235*	429	73
6	WP_054237237	*Actinobacteria bacterium OK006*	418	68
7	WP_051827886	*Streptomyces bicolor*	433	67
8	WP_035732764	*Frankia* sp. *Allo2*	419	71
9	WP_018961660	*Streptomyces* sp. *CNB091*	424	71
10	WP_051919510	*Streptomyces* sp. *NRRL F-5140*	424	68

**Table 3:** **Top 10 BlastP results for JadL against the TCDB**.

**Hit**	**Protein accession**	**Organism**	**Amino acid length**	**Identity**	**TC number**
1	D3Q871	*Stackebrandtia nassauensis (strain DSM 44728/NRRL B-16338/NBRC 102104/LLR-40K-21)*	417	31	2.A.1.21.11
2	Q7BKK4	*Streptococcus pneumoniae*	405	24	2.A.1.21.22
3	Q0E7C5	*Listonella anguillarum serovar O2*	437	25	2.A.1.38.2
4	Q9X4X4	*Pseudomonas abietaniphila*	547	25	2.A.1.30.1
5	H5X1B8	*Saccharomonospora marina XMU15*	395	27	2.A.1.21.21
6	O32859	*Mycobacterium fortuitum*	409	25	2.A.1.21.4
7	P95827	*Streptococcus pyogenes, and OS Streptococcus pneumoniae*	405	22	2.A.1.21.1
8	P64783	*Mycobacterium tuberculosis*	419	25	2.A.1.21.12
9	O31137	*Mycobacterium smegmatis*	419	23	2.A.1.21.3
10	A6QJ21	*Staphylococcus aureus (strain Newman)*	397	21	2.A.1.21.7

### Construction and confirmation of the *jadL* disruption mutant

*S. venezuelae* protoplasts were transformed with pJV106A bearing the *jadL* disruption cassette. After several rounds of sporulation to facilitate heterologous recombination, a double crossover mutant sensitive to thiostrepton and resistant to apramycin was confirmed to contain the disruption cassette by amplification of a 3 kb PCR product using primers flanking *jadL* (P_L-for_/P_L-rev_). This strain was designated *S. venezuelae* VS678. A corresponding 1.8 kb band was amplified from the wildtype strain (Figure 2). The orientation of the apramycin cassette was confirmed using PCR. Amplification was observed only when the primers pairs P_L-for/_P_Apr-for_ and P_L-rev/_P_Apr-rev_ were used indicating an opposite orientation of the apramycin resistance gene relative to *jadL* (Figure 2).

### Analysis and quantification of JdDS production in *jadL* disruption strain *S. venezuelae* VS678

**Figure 3:**
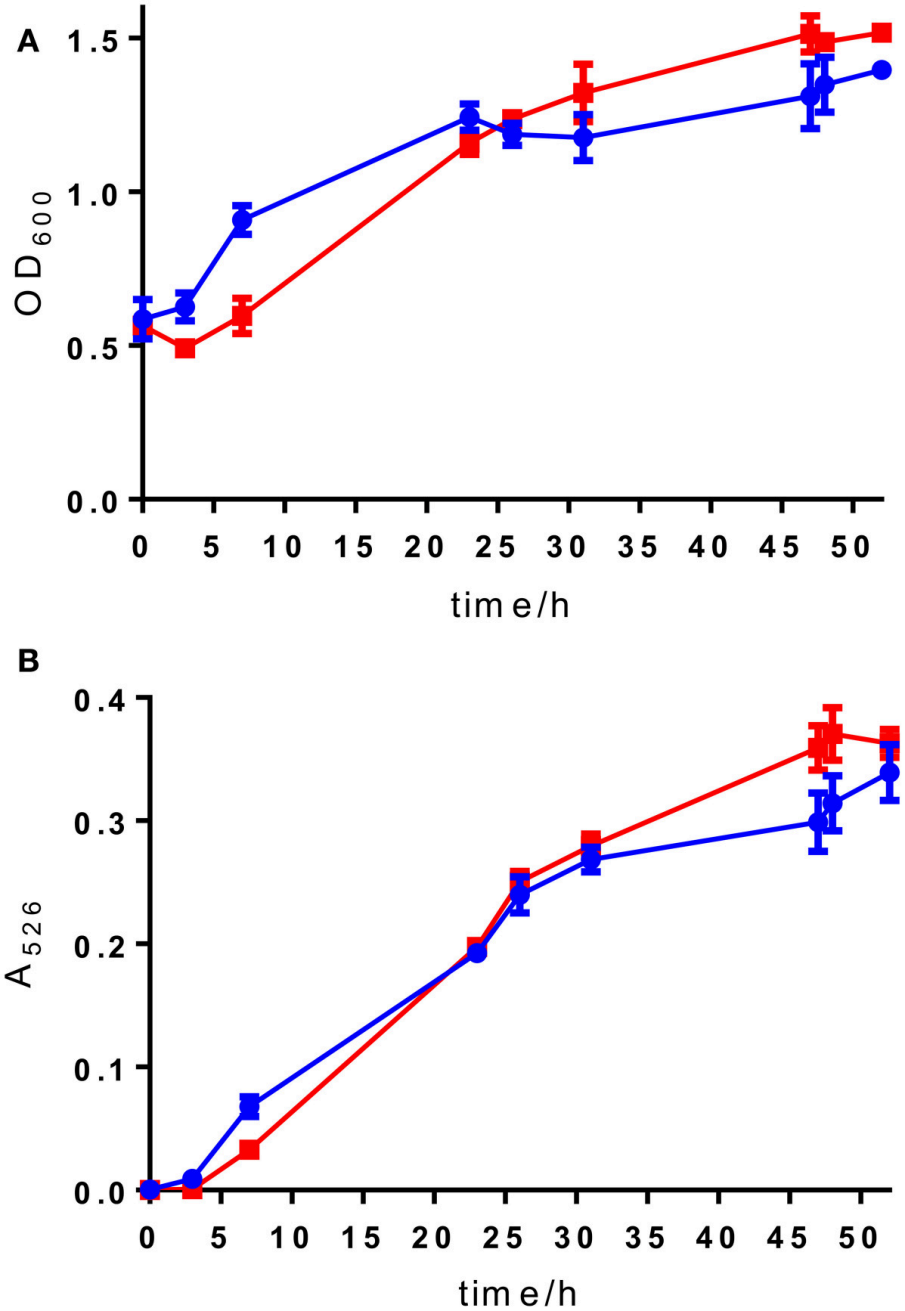
**(A)** OD_600_ readings and **(B)** A_526_ readings for wild type *S. venezuelae* ISP5230 (blue circles) and *S. venezuelae* VS678 (red squares). Error bars show the standard deviation and squares/circles shown the mean of the data from triplicate samples.

**Figure 4:**
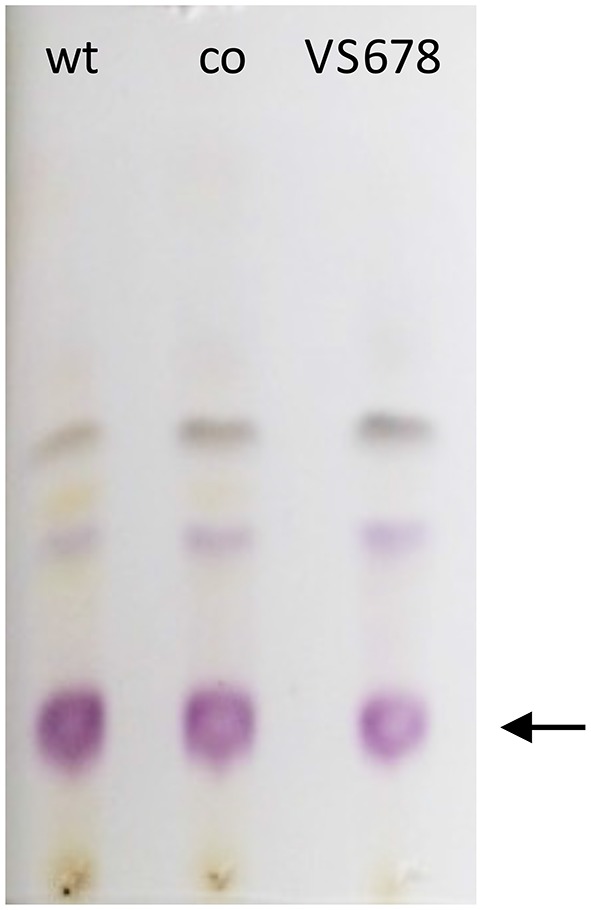
**TLC developed with 5:5:1 ethyl acetate:acetonitrile:water showing colored materials isolated from bacterial fermentations with ***S. venezuelae*** ISP5230 (wt) and ***S. venezuelae*** VS678 in the presence of **d**-serine**. Co refers to the co-spot where material from both wt and VS678 strains were spotted. **JdDS**, the major product, is indicated with an arrow.

**Figure 5:**
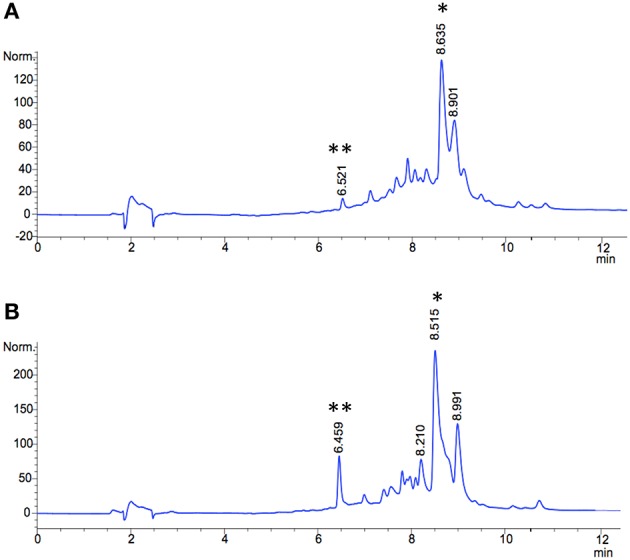
**HPLC traces of methanol extracts (silica-phenyl column) from jadomycin productions with D-serine (A)** wild type *S. venezuelae* ISP5230 **(B)** *S. venezuelae* VS678. The signal corresponding to **JdDS** is indicated by an asterisk (^*^), and **Cam** is indicated by a double asterics (^**^).

**Figure 6:**
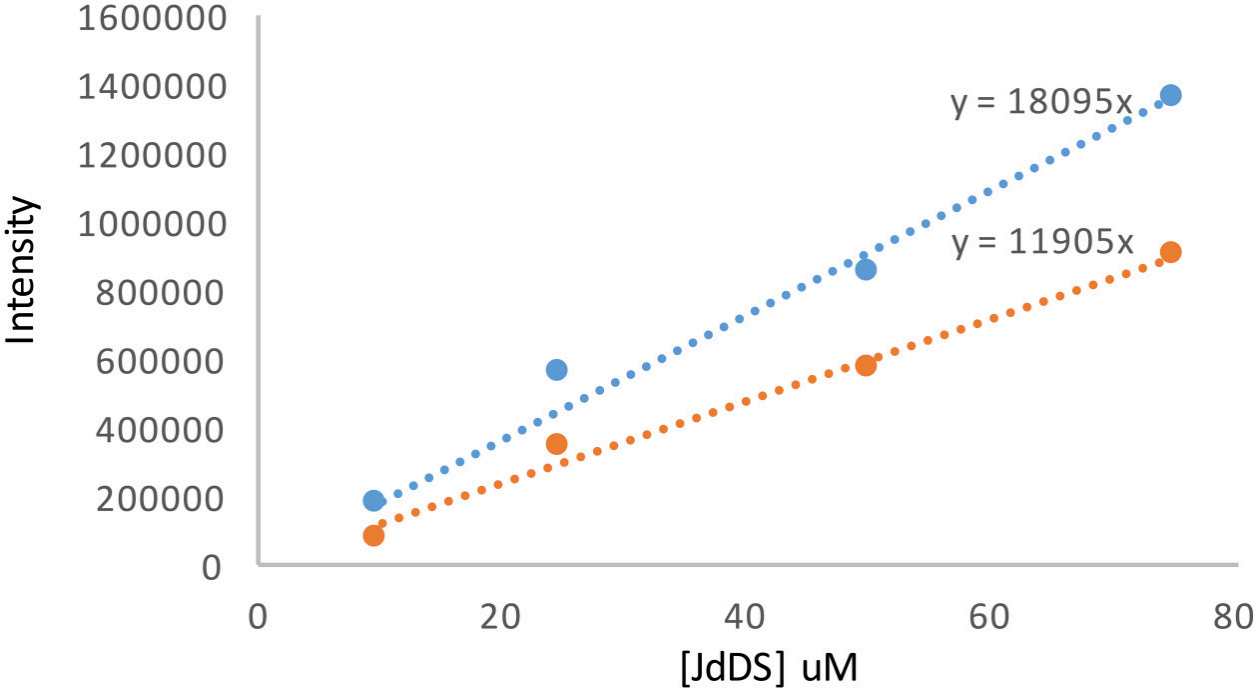
**Calibration curves for JdDS quantification using LC-MS^**2**^**. The blue line corresponds to the area beneath the parent ion [M+H]^+^ m/z 524 and the orange line to the area beneath the fragment ion m/z 394.

Selection of a single amino acid in the MSM culture media to produce a single E-ring variant was necessary to facilitate quantification. We selected d-serine as the amino acid because these culture conditions and the final jadomycin product, **JdDS**, are well characterized and extensively used as a standard in our laboratory (Robertson et al., 2016). After induction of jadomycin production by ethanol shock, growth curves for the disruption mutant and a wildtype control were monitored over 52 h (Figure 3). Cell growth in the disruption mutant cultures appeared to lag versus the wildtype over the first 24 h, but then caught up. The absorbance values at A_526_, that provide an estimate for excreted colored natural products, showed similar values for both strains after 52 h. Initial values were lower in the disruption mutant, consistent with the growth curve. In order to quantify the final amount of **JdDS** obtained, the methanol extract from the Si-Ph column was concentrated to dryness yielding 3.1 mg crude material from the wildtype strain and 2.8 mg from the disruption strain. TLC analysis showed that the colored compounds produced by both strains were identical (Figure 4), and that **JdDS** was produced by both strains. HPLC analysis showed the peak corresponding the **JdDS** at R_t_ 8.5 min in both crude samples (Figure 5). By mass spectral analysis, it was determined that 1.1 mg **JdDS** was produced by the wildtype and 1.3 mg was produced by the disruption mutant per 50 mL culture using the calibration curves shown in Figure 6. Our results clearly show, contrary to our hypothesis, that disruption of the MFS family protein *jadL* does not significantly affect jadomycin natural product production. Over 52 h, the disruption strain *S. venezuelae* VS678 grew comparatively to the wildtype and produced a similar final concentration of **JdDS**, by both by qualitative (HPLC, TLC) and quantitative (LCMS^2^) analysis. That jadomycin production was not obviated rules out the possibility of polar effects arising from the insertion of the apramycin disruption cassette. The methodology used for jadomycin isolation in which the first step involves removal of bacterial cells through pelleting ensures that only materials excreted from the cells (the supernatant) are collected. Therefore, we believe that our data shows that **JdDS** is being effectively excreted from cells of the disruption mutants. The initially depressed growth rates observed in the disruption strain may reflect stress induced from the disruption of JadL, but that the growth improves subsequently, suggest that alternate, currently unknown, mechanisms of **JdDS** export may be induced. Additionally, an increase of **Cam** levels in the HPLC trace of the crude isolate from the disruption mutant supports that jadomyicn biosynthesis may have been somewhat strained, resulting in the production of **Cam**. There have been a number of studies on the regulatory crosstalk between jadomycins and **Cam** biosynthesis in *Streptomyces venezuelae* ISP5230 (Fernandez-Martinez et al., 2014; Robertson et al., 2015, 2016; Sekurova et al., 2016).

### Discussion on the role of JadL in secondary metabolite production

Our data suggests that disruption of *jadL* does not have a substantive effect on on **JdDS** production. These results were rather unexpected, as we anticipated that without functional JadL, **JdDS** would accumulate within *S. venezuelae* and disrupt growth and/or reduce jadomycin production significantly. It is noteworthy that depressed growth rates were observed in the first 24 h, after which the growth of the disruption mutant recovered relative to growth of the wildtype. Presumably, alternate export mechanisms are induced during the first 24 h. The appearance of **Cam** in the disruption mutant may be an indication of additional stress on *S. venezuelae* VS678. It is entirely plausible that *S. venezuelae* possesses other transporters capable of exporting jadomycins, indicating JadL is not essential for this role. This is consistent with the role of the MFS transporter, SirA, present in the sirodesmin biosynthetic gene cluster in the fungus, *Leptosphaeria maculans* (Gardiner et al., 2005). SirA, whilst present within the biosynthetic gene cluster for sirodesmin, was determined not to be solely responsible for the efflux of endogenously produced sirodesmin, however, SirA did contribute toward self-protection. Our data showing the comparable growth for the wild-type and blocked mutant strains is suggestive that if JadL is responsible for self-resistance in *S. venzuelae* ISP5230, as was observed for SirA, that the concentrations of the jadomycin excreted into the media are insufficient to have a deleterious effect upon the growth of *S. venezuelae* ISP5230.

## Author contributions

SF and JM performed experiments. SF, JM, LV, and DJ conceived the work. SF and DJ prepared the manuscript.

## Funding

LV and DJ acknowledge funding from NSERC. DJ acknowledges funding from the NSHRF, NSERC, and CIHR. SF is a Killam Trust predoctoral scholar and a CRTP trainee funded in partnership with the Canadian Cancer Society, Nova Scotia Division.

### Conflict of interest statement

The authors declare that the research was conducted in the absence of any commercial or financial relationships that could be construed as a potential conflict of interest.
